# Optimal site for ultrasound-guided venous catheterisation in paediatric patients: an observational study to investigate predictors for catheterisation success and a randomised controlled study to determine the most successful site

**DOI:** 10.1186/s13054-014-0733-4

**Published:** 2015-01-20

**Authors:** Jun Takeshita, Yoshinobu Nakayama, Yasufumi Nakajima, Daniel I Sessler, Satoru Ogawa, Teiji Sawa, Toshiki Mizobe

**Affiliations:** Emergency and Critical Care Medicine, National Hospital Organization Kyoto Medical Center, 1-1 Fukakusamukaihata-cho Fushimi-ku, Kyoto, 612-8555 Japan; Department of Anaesthesiology and Intensive Care, 465 Kawaramachi Hirokoji Kamigyo-Ku, Kyoto, 602-8566 Japan; Department of Outcomes Research, 9500 Euclid Avenue, Cleveland, OH 44195 USA

## Abstract

**Introduction:**

Venous catheterisation in paediatric patients can be technically challenging. We examined factors affecting catheterisation of invisible and impalpable peripheral veins in children and evaluated the best site for ultrasound-guided catheterisation.

**Methods:**

Systolic pressure, age, sex, and American Society of Anaesthesiologists (ASA) physical status were determined in 96 children weighing less than 20 kg. Vein diameter and subcutaneous depth were measured with ultrasound. Logistic regression was used to evaluate the contribution of these factors to cannulation success with (n = 65) or without (n = 31) ultrasound guidance. Thereafter, we randomly assigned 196 patients for venous catheter insertion in the dorsal veins of the hand, the cephalic vein in the forearm, or the great saphenous vein. Success rates and vein diameters were evaluated by using Dunn tests; insertion time was evaluated by using Kaplan-Meier cumulative incidence analysis.

**Results:**

Independent predictors of catheterisation were ultrasound guidance (odds ratio (OR) = 7.3, 95% confidence interval (CI) 2.0 to 26.0, *P* = 0.002), vein diameter (OR = 1.5 per 0.1 mm increase in diameter, 95% CI 1.1 to 2.0, *P* = 0.007), and ASA physical status (OR = 0.4 per status 1 increase, 95% CI 0.2 to 0.9, *P* = 0.03). Cephalic veins were significantly larger (cephalic diameter 1.8 mm, *P* = 0.001 versus saphenous 1.5 mm, *P* <0.001 versus dorsal 1.5 mm). Catheterisation success rates were significantly better at the cephalic vein than either the dorsal hand or saphenous vein (cephalic 95%, 95% CI 89% to 100%, *P* <0.001 versus dorsal 69%, 95% CI 56% to 82%, *P* = 0.03 versus saphenous 75%, 95% CI 64% to 86%).

**Conclusions:**

The cephalic vein in the proximal forearm appears to be the most appropriate initial site for ultrasound-guided catheterisation in invisible and impalpable veins of paediatric patients.

**Trial registry number:**

UMIN Clinical Trials Registry as UMIN000010961. Registered on 14 June 2013.

## Introduction

Peripheral venous access is crucial for perioperative administration of drugs and fluids. In paediatric patients, the veins in the dorsal network of the hands or feet are generally selected for the initial catheterisation attempt since they are often visible or at least palpable. When veins are invisible or impalpable because of pre-operative dehydration or thick subcutaneous tissue, as is common in children from 1 to 3 years of age [1], peripheral venous catheterisation can be difficult and time-consuming.

Various techniques have been reported to aid catheterisation in such cases [2-5]. However, these methods (cut-downs, local warming, and nitroglycerin ointment) are rarely used, because they are time-consuming [5] or overly invasive [2,3]. Ultrasonography is an easy and non-invasive technique that has been used to guide peripheral venous access, similar to that for central venous access [6-8] and radial artery catheterisation [9-11].

Ultrasound guidance is reported to facilitate peripheral venous catheterisation in children and to have advantages over blind techniques, especially for challenging veins [1,12-14]. However, the factors contributing maximally to successful catheterisation remain unknown, as does the most suitable site for ultrasound-guided peripheral vein catheterisation [12,13].

In an initial observational study, we determined the factors that affect successful catheterisation of invisible and impalpable peripheral veins in paediatric patients weighing less than 20 kg. In a subsequent randomised trial, we determined the best site for ultrasound-guided catheterisation. Specifically, we tested the hypothesis that ultrasound-guided catheterisation is maximally successful at the cephalic vein in the straight portion of the proximal forearm (not including the antecubital fossa) than at dorsal hand veins or at the medial malleoli level for the great saphenous vein.

## Methods

Our study was approved by the Kyoto Prefectural University of Medicine Institutional Review Board, Kyoto, and registered at UMIN Clinical Trials Registry [15] as UMIN000010961 (14 June 2013; principal investigator, Toshiki Mizobe). Written informed consent was obtained from the guardians of all patients. For the present study, we enrolled paediatric patients (weight of less than 20 kg) who were admitted for elective surgery in the Kyoto Prefectural University of Medicine in 2013 and who had invisible and impalpable veins at all puncture sites. Patients with any prior venous catheterisation were excluded from the study.

Two Japanese Society of Anaesthesiologist Board-certified anaesthesiologists performed the peripheral venous catheterisations. Each had previously performed more than 50 ultrasound-guided peripheral venous catheterisations in paediatric patients. To assess the consistency of catheterisation time between the two, in a preliminary study, we quantified inter-operator reliability (intra-class correlation coefficient of more than 75%) [16,17]. Each anaesthesiologist performed ultrasound-guided peripheral venous catheterisations in 15 patients with similar clinical characteristics (vein diameter, subcutaneous vein depth, catheter size, puncture site, and so on), and the catheterisation time was evaluated. The intra-class correlation coefficient was 0.86 (95% confidence interval (CI) 0.66 to 0.96; *P* <0.01), indicating good reliability.

**Figure 1:**
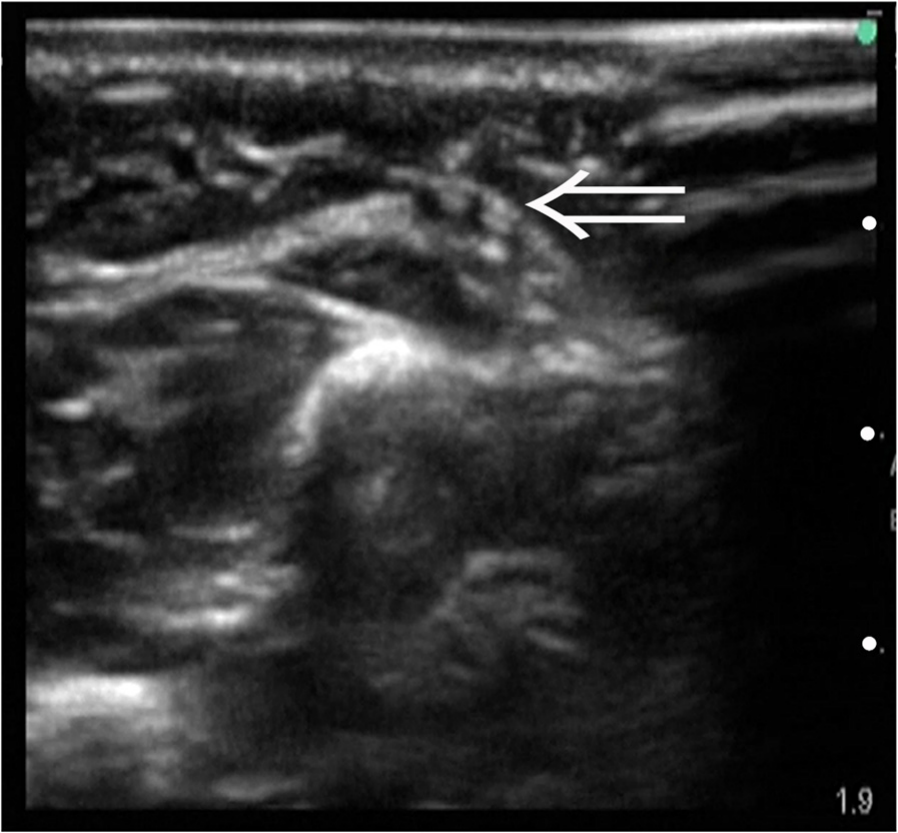
**Ultrasound-guided catheterisation for the cephalic vein in the proximal forearm.** The 24-gauge catheter tip was visualized in the centre of the vein (arrow). White dots indicate 5-mm increments.

Ultrasound guidance was performed as follows. We placed a tourniquet a few centimetres proximal to the puncture site. A roll was positioned under the puncture site to keep it parallel to the floor, and the extremity was taped to maintain optimal extension. After cleaning with povidone-iodine solution, a Sonosite M-turbo Ultrasound System (Fujifilm SonoSite Japan, Inc., Tokyo, Japan) with an SLAx/13-6 MHz transducer (hockey-stick type) in a sterile cover was positioned at the puncture site such that the vein was visualized in the short axis (out-of-plane approach). A catheter was inserted 1 to 2 mm distal to the transducer at a 10° to 30° puncture angle, adjusting the catheter tip toward the centre of the of the target vein until the anterior wall was seen to collapse on the display. The presence of blood was confirmed in the catheter hub, and the catheter was advanced slightly at a reduced angle in an effort to avoid the posterior wall of the vein, as shown in Figure 1.

Upon removing the stylet, if blood flow continued, we replaced the stylet and advanced the catheter into the vein, threading it off the needle; if the blood flow disappeared or when the puncture pressure completely collapsed the vein, we used a through-and-through approach. Specifically, we withdrew the stylet and then slowly withdrew the catheter tip until a flash of blood was visible in the catheter or hub. After confirmation of blood flow, we partially re-inserted the stylet to stiffen the cannula and then advanced the catheter into the vein.

Catheterisation without ultrasound guidance was blindly performed on the basis of normal venous anatomical location. No other methods (for example, bevel-down approach), materials (for example, guide wires), or local anaesthetic agents (for example, EMLA™ cream 5%, 600 Capability Green, Luton, UK. Penles tape 18mg, 1-5-22 Nakatsu, Kita-ku, Osaka, Japan) were used for any patient. We used a 24-gauge catheter (Jelco Plus, Smiths Medical Japan Ltd., Tokyo, Japan) for all catheterisation attempts. All catheterisations were performed during spontaneous breathing after slow induction with inhalation anaesthesia (4.0% to 5.0% sevoflurane).

### Observational study

Catheterisation in each patient was conducted at the cephalic vein in the straight portion of the proximal forearm (not including the antecubital fossa) or at the dorsal venous network of the hand, with or without ultrasound guidance, respectively. The site of intravenous catheterisation was chosen as follows. When the dorsal hand veins were neither visible nor palpable with tourniquet application, we performed catheterisation at the dorsal hand veins with or without ultrasound guidance alternately. When the dorsal hand veins were visible or palpable, we next assessed whether the cephalic veins were visible or palpable. When the cephalic veins were neither visible nor palpable, we conducted catheterisation only with ultrasound guidance because anatomically based blind cephalic vein catheterisation could injure the sensory branch of the radial nerve or the lateral antebrachial cutaneous nerve [18,19]. When the cephalic veins were visible or palpable, the patients were excluded from the observational study.

### Randomised trial

Considering the results of the abovementioned observational study, we hypothesized that catheterisation was more successful in veins with large diameters, such as cephalic veins. Specifically, our primary outcomes were the success rates for ultrasound-guided catheterisation of invisible and impalpable veins at three different anatomical sites. We also compared the catheterisation times as a secondary outcome.

At the pre- interview, we first assessed the visibility and palpability of veins at three different anatomical sites: the veins of the dorsal venous network of the hand, the cephalic vein in the straight portion of the proximal forearm (not including the antecubital fossa), and the great saphenous vein at the level of the medial malleoli. The judgments were carefully performed by two anaesthesiologists using tourniquet application. Patients were excluded from our study if any sites of these three veins were visible or palpable. Patients were also excluded if veins became visible or palpable after induction of general anaesthesia.

One hundred ninety-six patients were randomly divided into the three groups on the basis of computer-generated permuted blocks without stratification. Allocation was concealed in sequentially numbered opaque envelopes that were opened shortly before the induction of anaesthesia. Ultrasound-guided catheterisation was attempted at a single randomly allocated site.

### Measurements

We recorded the height, weight, age, sex, American Society of Anaesthesiologists (ASA) physical status, systolic and diastolic arterial pressure at the time of catheterisation, vein diameter, subcutaneous venous depth, puncture site, use of ultrasound guidance, insertion time, number of attempts (until successful insertion or until the attempt was abandoned), and overall catheterisation success rate.

Blood pressure was measured at the contralateral brachial artery (non-cannulated side) by using a blood pressure cuff immediately before the catheterisation attempt. Venous diameter was measured during two-dimensional imaging after the placement of a tourniquet and was considered the distance between the trailing and leading edges of the vein, as recommended by the American Society of Echocardiography [20], whereas venous depth was considered the distance from the transducer to the near edge of the vein.

All of the patients’ clinical and demographic measurements, including insertion times and ultrasound measurements, were performed by two anaesthesiologists other than the two investigators who performed the catheterisations. The two anaesthesiologists who performed the ultrasound measurements were board-certified anaesthesiologists who were well trained in ultrasound machine and had each conducted at least 50 ultrasound-guided peripheral venous catheterisations before the study started. Venous depth measurements were conducted without compressing tissues with the ultrasound probe. The results of the measurements were not shared with the investigators.

Catheterisation was considered complete when venous blood flow was confirmed after inserting the full length of the catheter. If extravasation was observed on injection, the attempt was considered a failure. When initial insertion failed, catheterisation was again attempted at the same vein. The number of attempts until catheterisation was completed was recorded, and more than three attempts were considered catheterisation failure. If the anaesthesiologist judged it imprudent to continue at the same vein (for example, because of hematoma), catheterisation was considered to have failed. Insertion duration was considered to be the time elapsed from initial skin puncture until catheterisation was completed or failed, with an upper limit of 3 minutes; attempts requiring more time were also considered catheterisation failures.

### Data analysis

The sample size estimate for the initial observational study was based on our previous study of ultrasound-guided radial arterial catheterisation in paediatric patients [21]. Specifically, we estimated that 92 patients would provide 80% power for detecting a 50% improvement in the success rate from 60% to 90% at an α-level of 0.05. Allowing for dropouts and technical problems, we therefore enrolled 96 patients in our observational study.

Multivariable logistic regression with the simultaneous method was used. The independent variables were age (months), sex (male/female), ASA physical status, systolic blood pressure (millimetres of mercury), vein diameter (millimetres), depth of the vein (millimetres), and ultrasound guidance (with/without). Overall catheterisation success was the dependent variable. Independent variables that were not normally distributed were logarithmically transformed.

The sample size for the randomised trial was based on 80% power to detect a difference in success rates from 65% to 95% (based on our observational results) at an α-level of 0.05. The minimum number of patients per group was 46, and we targeted approximately 50 patients per group to allow for dropouts and technical failures.

We used Kaplan-Meier cumulative incidence plots with log-rank tests to evaluate time to successful catheterisation in the three groups. Overall catheterisation success rates and vein diameters among the three groups were compared with Dunn tests.

Statistical analyses were performed with StatFlex version 6.0 (Artech Co., Ltd., Osaka, Japan) and SPSS 19.0 statistical package (SPSS, Chicago, IL, USA). Sample size was calculated by PASS 11 (NCSS, Kaysville, UT, USA) and Power and Sample Size Calculations version 3.0.43 (Vanderbilt Biostatistics, Nashville, TN, USA). The analyses were supervised by a trained statistician. Values are expressed as median (quartiles 1 to 3). A *P* value of less than 0.05 was considered statistically significant.

## Results

### Observational study

**Figure 2:**
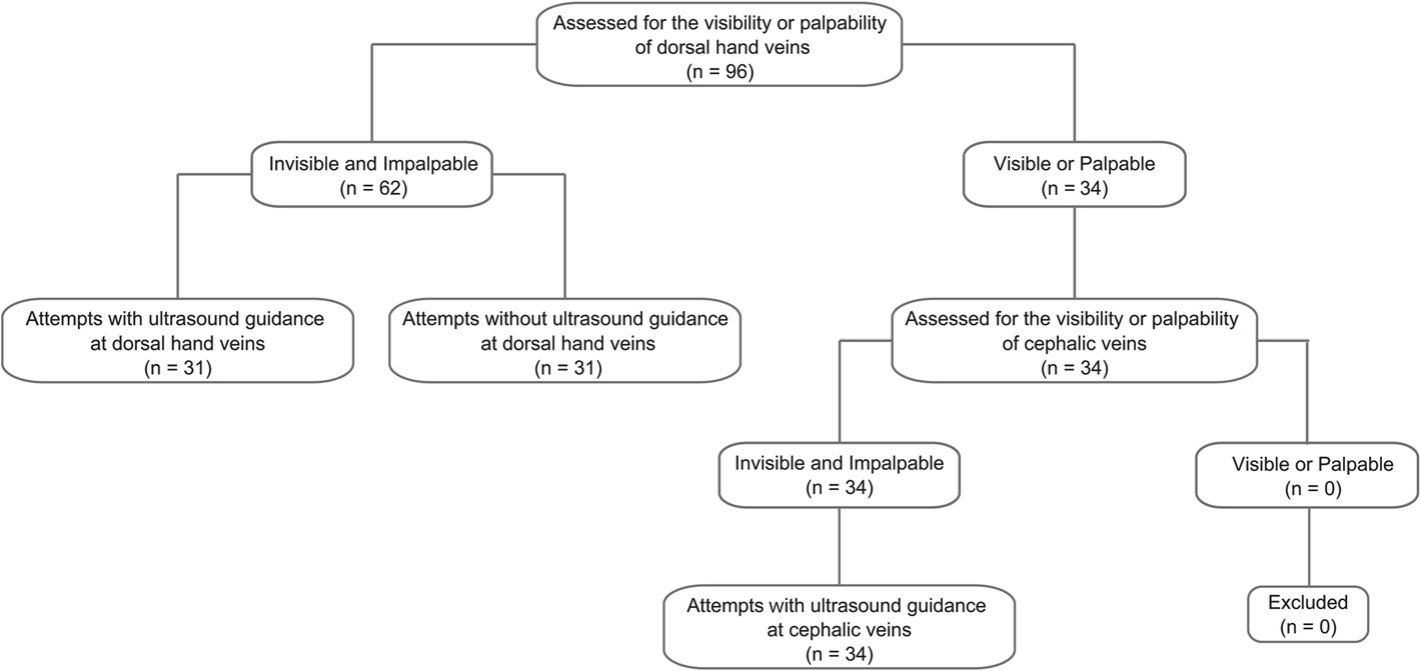
**Trial diagram for the observational study.**

Ninety-six patients were included in our observational study. The trial diagram is shown in Figure 2. No patient was excluded in this phase. The demographic characteristics of the patients are shown in Table 1. Age, weight, and height were highly multicollinear (r >0.80); therefore, we selected age as an independent variable representing all three characteristics. Similarly, systolic and diastolic arterial pressures were multicollinear (r >0.80); we therefore used systolic pressure as the independent variable in logistic regression analysis.

**Table 1:** **Demographic characteristics of 96 patients included in the multiple logistic regression analysis**

**Parameters**	**Values**
Age, months	22 (13-34)
Sex, male	66
Height, cm	80 (72-92)
Weight, kg	11 (9-13)
Venous depth at puncture site, mm	1.8 (1.4-2.7)
Diameter of target vein, mm	1.4 (1.2-1.7)
Puncture site, dorsal/cephalic	62/34
Systolic blood pressure, mm Hg	90 (82-102)
Diastolic blood pressure, mm Hg	47 (40-55)
With ultrasound guidance	65
Trisomy 21	3
ASA-PS	
PS 1	64
PS 2	15
PS 3	16
PS 4	1
PS 5	0
Catheterisation time, s	43 (25-180)
Number of trials, times	1 (1-1)
Total success rate, %	66.7

Cephalic refers to the cephalic veins in the proximal forearm. Dorsal refers to the veins of the dorsal venous network of the hand. ASA-PS, the American Society of Anaesthesiologists physical status.

The results of logistic regression analysis are shown in Table 2. Significant predictors of catheterisation success were ultrasound guidance (odds ratio (OR) = 7.3, 95% CI 2.0 to 26.0, *P* = 0.002), target vein diameter (OR = 1.5 per 0.1 mm increase in diameter, 95% CI 1.1 to 2.0, *P* = 0.007), and ASA physical status (OR = 0.4 per status 1 increase, 95% CI 0.2 to 0.9, *P* = 0.03). When the incidence of an outcome of interest is frequent (>10%) in the study population, the OR estimated by logistic regression analysis overestimates the risk ratio (RR) [22-24]. Therefore, we calculated the RR for ultrasound guidance (RR = 2.3, 95% CI 1.4 to 3.8, *P* = 0.002).

**Table 2:** **Factors affecting the overall success rate of venous catheterisation extracted with multiple logistic regression analysis (n = 96)**

**Independent variables**	**β**	**Odds ratio**	**95% confidence interval**	***P*** **value**
With ultrasound guidance	1.98	7.3	2.0-26.0	0.002
Venous diameter, per 0.1 mm	0.38	1.5	1.1-2.0	0.007
ASA-PS, per status 1	−0.86	0.4	0.2-0.9	0.03
Age, months		0.9		0.08
Sex, male/female		2.6		0.2
Systolic blood pressure, mm Hg		1.0		0.06
Depth of the vein, mm		0.7		0.42

Area under the curve = 0.88. β = partial regression coefficient. ASA-PS, American Society of Anaesthesiologists physical status.

The demographic characteristics of the three individual groups included in the observational study are shown in Table 3. Catheterisation success rates were significantly greater at the cephalic vein with ultrasound guidance than at the dorsal hand with ultrasound guidance or without ultrasound guidance (cephalic vein with ultrasound guidance: 97%, 95% CI 77% to 100%, *P* = 0.02 versus dorsal hand with ultrasound guidance: 68%, 95% CI 47% to 81%, *P* <0.001 versus dorsal hand without ultrasound guidance: 39%, 95% CI 24% to 58%). The time required for successful catheterisation was significantly shorter at the cephalic vein with ultrasound guidance than at the dorsal hand vein with ultrasound guidance or without ultrasound guidance (cephalic vein with ultrasound guidance: 23 seconds, *P* <0.001 versus dorsal hand vein with ultrasound guidance: 54 seconds, *P* <0.001 versus dorsal hand vein without ultrasound guidance: 180 seconds). Furthermore, the diameter of the cephalic vein was significantly greater than that of the dorsal hand vein (cephalic veins 1.8 mm, *P* = 0.002 versus dorsal veins 1.4 mm) in patients undergoing ultrasound-guided venous catheterisation.

**Table 3:** **Demographic characteristics of three individual groups in the observational study**

**Parameters**	**Cephalic with ultrasound guidance**	**Dorsal hand with ultrasound guidance**	**Dorsal hand without ultrasound guidance**
	**(n = 34)**	**(n = 31)**	**(n = 31)**
Age, months	25 (14-40)	23 (12-37)	21 (10-34)
Sex, male	23	22	21
Height, cm	83 (73-95)	79 (73-91)	77 (67-92)
Weight, kg	11 (9-14)	10 (9-13)	10 (8-12)
Venous depth, mm	2.9 (2.2-3.6)^a,b^	1.7 (1.5-2.1)^b^	1.4 (1.1-1.8)
Diameter of target vein, mm	1.8 (1.4-2.0)^a,b^	1.4 (1.2-1.6)	1.4 (1.1-1.5)
Systolic blood pressure, mm Hg	93 (84-99)	90 (81-98)	89 (82-97)
Diastolic blood pressure, mm Hg	46 (41-54)	45 (40-55)	43 (38-52)
Trisomy 21	0	2	1
ASA-PS			
PS 1, n = 64	23	20	21
PS 2, n = 15	5	5	5
PS 3, n = 16	6	6	4
PS 4, n = 1	0	0	1
PS 5, n = 0	0	0	0
Number of trials, times	1 (1-1)	1 (1-1)	1 (1-1)
Success rates, %	97^a,b^	68^b^	39
Catheterisation times, s	23 (18-33)^a,b^	54 (31-180)^b^	180 (50-180)

Cephalic refers to cephalic vein in the straight portion of the proximal forearm. Dorsal hand refers to the veins of the dorsal venous network of the hand. ^a^ *P* <0.05 versus Dorsal hand vein with ultrasound guidance. ^b^ *P* <0.05 versus Dorsal hand vein without ultrasound guidance. ASA-PS, the American Society of Anaesthesiologists physical status.

### Randomised trial

**Figure 3:**
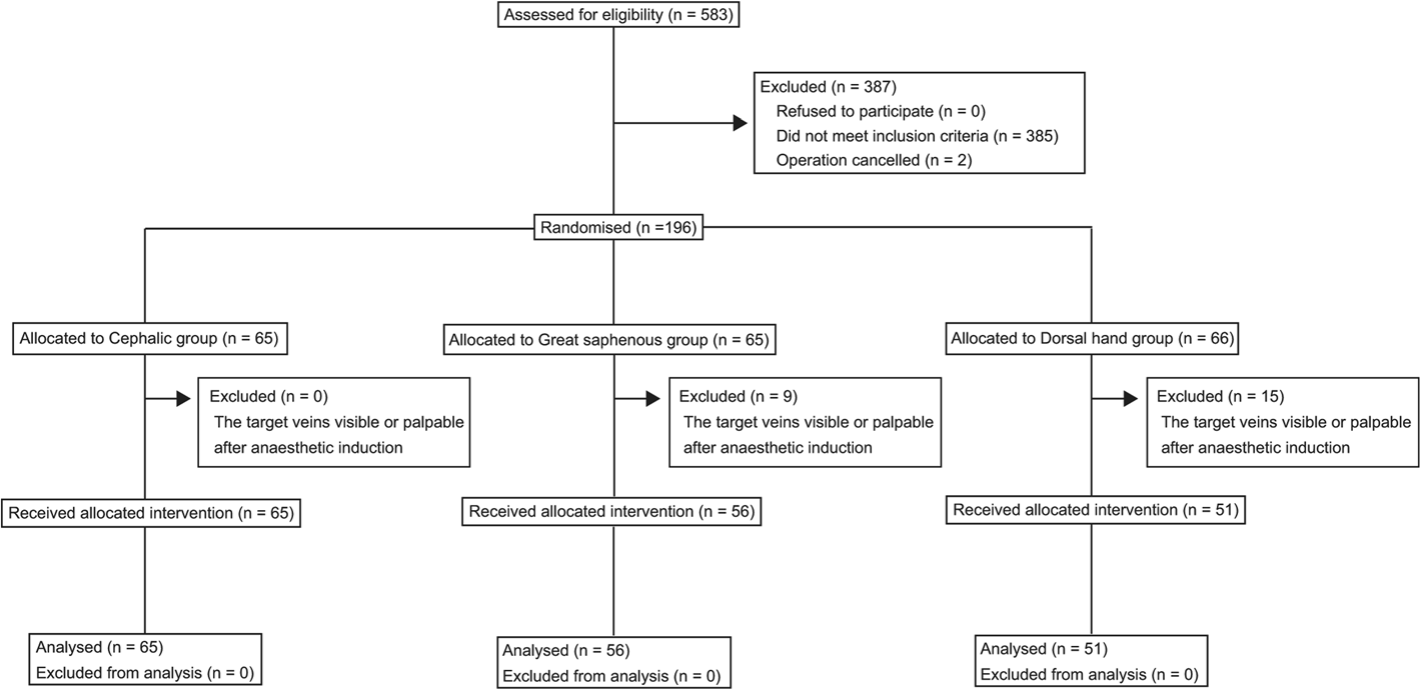
**Trial diagram for the randomised trial.**

The diagram for the randomised trial is shown in Figure 3. Twenty-four patients were excluded after randomisation because the target veins were visible or palpable after anaesthetic induction. The clinical and demographic characteristics of the 172 participants are shown in Table 4. The cephalic vein in the proximal forearm had the largest diameter among the three veins: cephalic veins 1.8 mm, *P* = 0.001 versus great saphenous veins 1.5 mm; *P* <0.001 versus dorsal hand veins 1.5 mm; great saphenous veins, *P* >0.99 versus dorsal hand veins.

**Table 4:** **Demographic characteristics of patients enrolled in the prospective randomised trial (n = 172)**

**Parameters**	**Cephalic**	**Great saphenous**	**Dorsal hand**
	**(n = 65)**	**(n = 56)**	**(n = 51)**
Age, months	20 (9-31)	21 (8-34)	24 (13-38)
Sex, male	41	34	38
Height, cm	78 (68-87)	77 (66-88)	81 (71-91)
Weight, kg	11 (8-13)	10 (8-13)	10 (8-13)
Venous depth, mm	3.6 (2.8-4.4)^a,b^	2.4 (1.9-2.8)^b^	1.6 (1.1-2.5)
Diameter of target vein, mm	1.8 (1.5-2.1)^a,b^	1.5 (1.3-1.7)	1.5 (1.2-1.7)
Systolic blood pressure, mm Hg	90 (84-100)	95 (83-102)	87 (82-96)
Diastolic blood pressure, mm Hg	46 (40-54)	48 (42-55)	43 (40-54)
ASA-PS			
PS 1, n = 94	37	30	27
PS 2, n = 23	9	8	6
PS 3, n = 52	18	17	17
PS 4, n = 3	1	1	1
PS 5, n = 0	0	0	0
Number of trials, times	1 (1-1)	1 (1-1)	1 (1-1)
Success rates, %	95^a,b^	75	69

Cephalic refers to the cephalic vein in the straight portion of the proximal forearm. Dorsal hand refers to the veins of the dorsal venous network of the hand. Great saphenous refers to the great saphenous veins at the ankle. ^a^ *P* <0.05 versus great saphenous vein. ^b^ *P* <0.05 versus dorsal hand vein. ASA-PS, the American Society of Anaesthesiologists physical status.

**Figure 4:**
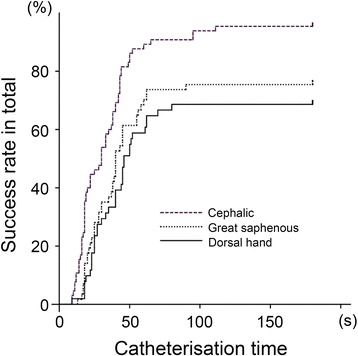
**Kaplan-Meier curves for catheterisation success time as a function of a puncture site.** Ultrasound-guided catheterisation of the cephalic vein in the proximal forearm had the shortest catheterisation time (cephalic, *P* <0.001 versus saphenous and dorsal; saphenous, *P* = 0.31 versus dorsal, log-rank test). The median catheterisation times for the three groups were as follows: the cephalic group, 30 seconds; the saphenous group, 40 seconds; and the dorsal group, 47 seconds.

Catheterisation success rates were significantly better at the cephalic vein than at either the dorsal hand or saphenous vein (cephalic: 95%, 95% CI 89% to 100%, *P* <0.001 versus dorsal hand: 69%, 95% CI 56% to 82%, *P* = 0.03 versus saphenous: 75%, 95% CI 64% to 86%). In contrast, the difference in success rates did not differ significantly between the dorsal hand and saphenous veins. The time required for successful catheterisation was significantly shorter at the cephalic vein than at the dorsal hand or great saphenous vein (*P* <0.001 for each); in contrast, insertion times did not significantly differ at the dorsal hand or saphenous veins (Figure 4).

## Discussion

In our observational analysis, catheterisation with ultrasound guidance, larger target vein diameter, and lower ASA physical status were associated with successful catheterisation of invisible and impalpable peripheral veins in paediatric patients weighing less than 20 kg. In the randomised trial, the cephalic vein in the proximal forearm had the highest success rate and required the least time for ultrasound-guided peripheral venous catheterisation than either of the other two insertion sites. Importantly, the diameter of the cephalic vein in the proximal forearm was greater than that of either the great saphenous vein at the ankle or the dorsal hand vein, supporting the findings of the observational study.

Our results are generally consistent with previous studies evaluating suitable sites for ultrasound-guided peripheral venous catheterisation in children [12,13]. In a recent small case series, catheterisation was successful in the great saphenous and the cephalic veins in the forearm [12]. Another observational study demonstrated more than 95% success rates using the great saphenous vein [13], which is another attractive cannulation site because the antecubital and saphenous veins are larger than hand veins [25]. Our study extends previous work by focusing on only invisible and impalpable peripheral veins; our results from a large observational and randomised population show that the most suitable site for ultrasound-guided catheterisation is the cephalic vein of the proximal forearm.

Our observational analysis revealed the relationships between successful catheterisation and the target vein diameter, ASA physical status, and catheterisation with ultrasound guidance. However, no association with subcutaneous venous depth was observed. This is in distinct contrast to ultrasound-guided radial artery catheterisation in paediatric patients where subcutaneous arterial depth between 2 and 4 mm has been strongly associated with successful catheterisation, with saline injection to augment depth improving success rates [21]. Possibly, diameter of vessels was more important than subcutaneous depth in venous catheterisation because veins are more compressible than arteries.

Compared with previous results concerning great saphenous veins [13], our study showed a lower success rate for this puncture site. This was possibly because our sample population included patients in whom catheterisation was more difficult than in the previous study’s subjects; 33% of our included subjects had an ASA physical status of at least 3. In other words, catheterisation attempts to the cephalic vein in the proximal forearm had a higher success rate regardless of the ASA physical status.

Catheterisation of the cephalic vein in the straight portion of the proximal forearm is not only easier and faster than at other sites but also less likely to suffer kinking resulting from patient movement. The great saphenous vein is further disadvantaged because it is difficult to detect extravasation at this site since it is often unavailable for intra-operative inspection. We thus conclude that the cephalic vein in the proximal forearm is the most appropriate initial site for ultrasound-guided catheterisation attempts.

The sensory branch of the radial nerve emerges proximally at an average distance of 8 cm (range of 6 to 11 cm) from the styloid process of the radius in adults [18]. Some have therefore suggested that cephalic vein catheterisation be performed at least 12 cm above the styloid process of the radius in adults. A study of adult cadavers demonstrated that the lateral antebrachial cutaneous nerve travels in close proximity to the cephalic vein in the proximal forearm, indicating that cephalic vein catheterisation in the proximal forearm might lead to lateral antebrachial cutaneous nerve injury [19].

The saphenous nerve travels along the saphenous vein, splitting into numerous branches [26]. At the ankle, the great saphenous vein lies close to the saphenous nerve or below the nerve; thus, venepuncture of the great saphenous vein at the ankle also carries a risk of nerve injury. Nerve injuries of the dorsal sensory branches of the hand during venepuncture have also been reported in adults [27]. It remains unclear whether these findings can be applied to paediatric patients. No nerve injuries were observed among our 268 patients; furthermore, we are unaware of reports of nerve injury resulting from either cephalic or saphenous vein catheterisation in paediatric patients.

It remains unclear whether an in-plane or out-of-plane approach is preferable for peripheral vascular catheterisation [28-30]. We used the out-of-plane approach because the in-plane approach can produce slice-thickness artefacts which might diminish the benefit of ultrasound guidance [21,31]. Furthermore, a 24-gauge catheter can appear to be in the same plane as a thin target vein on in-plane views, even in unsuccessful catheterisation.

We generally threaded the intravenous catheter directly off the insertion needle. The through-and-through technique was used only as a backup approach because it often produces a hematoma beyond the vein’s posterior wall, which can compromise subsequent catheterisation attempts should one prove necessary. The benefits [32-34] and difficulties [35] of other techniques to facilitate vessel catheterisation, including using guide-wires and the bevel-down approach, are well verified in adults but remain poorly evaluated in paediatric patients [33]. Ultrasound-guided peripheral venous catheterisation may be more successful when combined with these ancillary techniques.

Our study was conducted at a single centre by well-trained sonographers. Therefore, the results might not be directly applicable to other populations. Another limitation is that we used a small hockey-stick transducer, which provides high resolution. Other types of transducers may not work so well.

## Conclusions

In our initial observational analysis, ultrasound guidance, vein diameter, and ASA physical status were independent predictors of catheterisation of invisible and impalpable peripheral veins in paediatric patients weighing less than 20 kg. In our subsequent randomised trial, the cephalic vein in the proximal forearm had the highest success rate and required the least time for ultrasound-guided peripheral venous catheterisation as compared with the great saphenous vein at the ankle and the dorsal hand vein. Importantly, the diameter of the cephalic vein in the proximal forearm was greater than that of the other two veins examined in this study. The cephalic vein in the proximal forearm thus appears to be the most appropriate initial site for ultrasound-guided catheterisation in paediatric patients.

## Key messages

- Ultrasound guidance, vein diameter, and ASA physical status are independent predictors of catheterisation of invisible and impalpable peripheral veins in paediatric patients weighing less than 20 kg.
- The cephalic vein in the proximal forearm had the highest success rate and required the least time for ultrasound-guided peripheral venous catheterisation as compared with the great saphenous vein at the ankle and the dorsal hand vein.
- In paediatric patients weighing less than 20 kg, the diameter of the cephalic vein in the proximal forearm was greater than that of the great saphenous vein at the ankle and the dorsal hand vein.
- The cephalic vein in the proximal forearm appears to be the most appropriate site for the first attempt of ultrasound-guided catheterisation in paediatric patients.

## Abbreviations

ASA: American Society of Anaesthesiologists
CI: Confidence interval
OR: Odds ratio
RR: Risk ratio

## References

[1] Nafiu OO, Burke C, Cowan A, Tutuo N, Maclean S, Tremper KK (2010). Comparing peripheral venous access between obese and normal weight children. Paediatr Anaesth.

[2] Kissoon N, Frewen TC (1987). Pediatric venous cutdowns: utility in emergency situations. Pediatr Emerg Care.

[3] Vaksmann G, Rey C, Breviere GM, Smadja D, Dupuis C (1987). Nitroglycerine ointment as aid to venous cannulation in children. J Pediatr.

[4] Goren A, Laufer J, Yativ N, Kuint J, Ben Ackon M, Rubinshtein M (2001). Transillumination of the palm for venipuncture in infants. Pediatr Emerg Care.

[5] Lenhardt R, Seybold T, Kimberger O, Stoiser B, Sessler DI (2002). Local warming and insertion of peripheral venous cannulas: single blinded prospective randomised controlled trial and single blinded randomised crossover trial. BMJ.

[6] Hosokawa K, Shime N, Kato Y, Hashimoto S (2007). A randomized trial of ultrasound image-based skin surface marking versus real-time ultrasound-guided internal jugular vein catheterization in infants. Anesthesiology.

[7] Skippen P, Kissoon N (2007). Ultrasound guidance for central vascular access in the pediatric emergency department. Pediatr Emerg Care.

[8] Breschan C, Platzer M, Likar R (2009). Central venous catheter for newborns, infants and children. Anaesthesist.

[9] Schwemmer U, Arzet HA, Trautner H, Rauch S, Roewer N, Greim CA (2006). Ultrasound-guided arterial cannulation in infants improves success rate. Eur J Anaesthesiol.

[10] Shiver S, Blaivas M, Lyon M (2006). A prospective comparison of ultrasound-guided and blindly placed radial arterial catheters. Acad Emerg Med.

[11] Ueda K, Puangsuvan S, Hove MA, Bayman EO (2013). Ultrasound visual image-guided vs Doppler auditory-assisted radial artery cannulation in infants and small children by non-expert anaesthesiologists: a randomized prospective study. Br J Anaesth.

[12] Samoya SW (2010). Real-time ultrasound-guided peripheral vascular access in pediatric patients. Anesth Analg.

[13] Triffterer L, Marhofer P, Willschke H, Machata AM, Reichel G, Benkoe T (2012). Ultrasound-guided cannulation of the great saphenous vein at the ankle in infants. Br J Anaesth.

[14] Benkhadra M, Collignon M, Fournel I, Oeuvrard C, Rollin P, Perrin M (2012). Ultrasound guidance allows faster peripheral IV cannulation in children under 3 years of age with difficult venous access: a prospective randomized study. Paediatr Anaesth.

[15] UMIN-CTR Clinical Trial. https://upload.umin.ac.jp/cgi-open-bin/ctr/ctr.cgi?function=brows&action=brows&type=summary&recptno=R000012830&language=J.

[16] Landis JR, Koch GG (1977). The measurement of observer agreement for categorical data. Biometrics.

[17] Cicchetti DV, Sparrow SA (1981). Developing criteria for establishing interrater reliability of specific items: applications to assessment of adaptive behavior. Am J Ment Defic.

[18] Vialle R, Pietin-Vialle C, Cronier P, Brillu C, Villapadierna F, Mercier P (2001). Anatomic relations between the cephalic vein and the sensory branches of the radial nerve: How can nerve lesions during vein puncture be prevented?. Anesth Analg.

[19] Wongkerdsook W, Agthong S, Amarase C, Yotnuengnit P, Huanmanop T, Chentanez V (2011). Anatomy of the lateral antebrachial cutaneous nerve in relation to the lateral epicondyle and cephalic vein. Clin Anat.

[20] Lang RM, Bierig M, Devereux RB, Flachskampf FA, Foster E, Pellikka PA (2005). Recommendations for chamber quantification: a report from the American Society of Echocardiography’s Guidelines and Standards Committee and the Chamber Quantification Writing Group, developed in conjunction with the European Association of Echocardiography, a branch of the European Society of Cardiology. J Am Soc Echocardiogr.

[21] Nakayama Y, Nakajima Y, Sessler DI, Ishii S, Shibasaki M, Ogawa S (2014). A novel method for ultrasound-guided radial arterial catheterization in pediatric patients. Anesth Analg.

[22] Zhang J, Yu KF (1998). What’s the relative risk? A method of correcting the odds ratio in cohort studies of common outcomes. JAMA.

[23] Zou G (2004). A modified poisson regression approach to prospective studies with binary data. Am J Epidemiol.

[24] Prasad K, Jaeschke R, Wyer P, Keitz S, Guyatt G, Evidence-Based Medicine Teaching Tips Working G (2008). Tips for teachers of evidence-based medicine: understanding odds ratios and their relationship to risk ratios. J Gen Intern Med.

[25] Riera A, Langhan M, Northrup V, Santucci K, Chen L (2011). Remember the saphenous: ultrasound evaluation and intravenous site selection of peripheral veins in young children. Pediatr Emerg Care.

[26] Wilmot VV, Evans DJ (2013). Categorizing the distribution of the saphenous nerve in relation to the great saphenous vein. Clin Anat.

[27] Horowitz SH (1994). Peripheral nerve injury and causalgia secondary to routine venipuncture. Neurology.

[28] Stone MB, Moon C, Sutijono D, Blaivas M (2010). Needle tip visualization during ultrasound-guided vascular access: short-axis vs long-axis approach. Am J Emerg Med.

[29] Mahler SA, Wang H, Lester C, Skinner J, Arnold TC, Conrad SA (2011). Short- vs long-axis approach to ultrasound-guided peripheral intravenous access: a prospective randomized study. Am J Emerg Med.

[30] Berk D, Gurkan Y, Kus A, Ulugol H, Solak M, Toker K (2013). Ultrasound-guided radial arterial cannulation: long axis/in-plane versus short axis/out-of-plane approaches?. J Clin Monit Comput.

[31] Goldstein A, Madrazo BL (1981). Slice-thickness artifacts in gray-scale ultrasound. J Clin Ultrasound.

[32] Mangar D, Thrush DN, Connell GR, Downs JB (1993). Direct or modified Seldinger guide wire-directed technique for arterial catheter insertion. Anesth Analg.

[33] Yildirim V, Ozal E, Cosar A, Bolcal C, Acikel CH, Kilic S (2006). Direct versus guidewire-assisted pediatric radial artery cannulation technique. J Cardiothorac Vasc Anesth.

[34] Ohara Y, Nakayama S, Furukawa H, Satoh Y, Suzuki H, Yanai H (2007). Use of a wire-guided cannula for radial arterial cannulation. J Anesth.

[35] Gerber DR, Zeifman CW, Khouli HI, Dib H, Pratter MR (1996). Comparison of wire-guided and nonwire-guided radial artery catheters. Chest.

